# Research on an islanding detection method suitable for distributed generation grid-connection complex system

**DOI:** 10.1371/journal.pone.0350272

**Published:** 2026-05-28

**Authors:** Wen Sun, Sihan Yu, Zhengye Jiang

**Affiliations:** 1 School of Electrical and Electronic Engineering, Anhui Science and Technology University, Bengbu, China; 2 China Triumph International Engineering Co., Ltd, Shanghai, China; State Grid Corporation of China, CHINA

## Abstract

With the increase in penetration rate of distributed generation under dual-carbon goals, hazards of unplanned islanding caused by grid-connection of distributed generation systems have attracted attention. Due to the dilution effect of characteristic parameters in multi-machine grid-connected systems, active and passive hybrid islanding detection is prone to have islanding detection blind zones. Therefore, an islanding detection method suitable for complex multi-machine grid-connected systems of distributed generation is proposed. To verify the feasibility of the proposed islanding detection method, a simulation model of the distributed generation grid-connected system was built in MATLAB/Simulink based on IEC 61850-7-420 standard. Two different operating conditions of islanding and grid-connection, were simulated to obtain voltage and current waveforms of each node, which were converted into time series. Then, time-frequency spectrum analysis and calculation were performed based on Short-Time Fourier Transform (STFT) to generate time-frequency spectrum. After feature extraction, training iterations were carried out using Convolutional Neural Network (CNN) algorithm to obtain the optimal islanding detection model. Simulation results show that the proposed method achieves a detection accuracy of 99.84% on the independent test set, with a missed detection rate of 3.4% under five-fold cross-validation, which significantly reduces the non-detection zone.

## Introduction

Against advancing background of dual-carbon goals, penetration rate of distributed generation continues to increase. This trend has significantly enhanced the complexity and uncertainty of power system networks, The islanding effect generated during grid-connection process of distributed generation systems has thus become a research issue [1]. Islanding effect refers to the phenomenon where distributed generation system continues to supply power to regional loads after being disconnected from grid [2]. As a large number of power electronic devices make power grid exhibit weak grid characteristics. When a distributed generation unit is unintentionally disconnected from the public power grid, it is of critical importance to achieve rapid and accurate islanding detection [3].

Hybrid islanding detection methods have currently become the mainstream detection methods for islanding detection in distributed generation grid-connected systems [4]. This method integrates the advantages of passive and active detection, adopting the collaborative strategy of passive detection as mainstay and active detection as supplement. This can effectively narrow islanding detection blind zone existing in passive detection while reducing the impact of active detection on power quality [5]. However, the performance of hybrid islanding detection methods is highly dependent on load characteristics and distributed generation characteristics, which still has detection blind zones. Specifically, it manifests as problems such as multi-criterion conflicts, abnormal responses to active disturbances, edge effects of detection blind zones, and threshold wandering zones, among which power balance is a particularly prominent situation [6]. When the output power of distributed generation sources is nearly completely matched with local load power, passive detection technologies cannot achieve effective identification due to weak parameter changes. At the same time, the interference signals injected by active detection may be absorbed by the impedance characteristics of loads, resulting in the inability of interference to cause detectable parameter deviations. In this case, the system may miss islanding determination because there is no abnormality in passive detection and no response in active detection. Moreover, in-phase fluctuation superposition of multiple distributed generation sources is likely to trigger high-frequency actions due to misjudgment of passive islanding detection, which in turn leads to unnecessary disconnection of micro-sources and generates the risk of misjudgment. Anti-phase fluctuation superposition may result in weak changes in characteristic parameters, with hidden dangers of missed islanding determination [7,8]. Furthermore, when active detection methods are used to inject interference signals, the in-phase superposed interference may exceed the limit on total harmonic distortion specified in the grid standard IEEE 1547 [9], thereby adversely affecting power quality of grid.

However, the essence of feature dilution is a decrease in the signal-to-noise ratio of individual features, without destroying the inherent correlation among multiple weak features. In view of detection blind zones in hybrid islanding detection method, an important solution is to introduce intelligent algorithms. Intelligent algorithms can accurately capture weak features in blind zones to improve detection accuracy. Especially in the case of integrating multi-parameter modeling, the islanding state can be accurately detected according to different situations, reducing the rates of false judgment and missed judgment. Due to the development and complexity of smart grid, it is imperative to study an accurate islanding detection technique. Currently, many researchers have proposed classification-based machine learning algorithms such as neural networks, support vector machines (SVM), and long short-term memory (LSTM) networks, which show great potential in improving the accuracy of islanding detection [10–14].

As shown in Table 1, relevant studies [15–19] indicate that existing intelligent algorithm-based islanding detection methods have achieved remarkable progress in detection speed and accuracy, effectively compensating for the inherent defects of traditional passive and active detection methods, yet there remains room for improvement in threshold adaptability and robustness under complex scenarios. The research introduces intelligent learning models such as LSTM, DNN, Random Forest, and KNN to replace the traditional fixed-threshold judgment logic, with improved robustness and anti-interference capability. However, such methods generally rely on discrete Fourier transform, symmetrical component extraction, wavelet transform, and other techniques to extract electrical features including voltage or current time-series components, harmonics, and phasor angles. Under complex operating conditions such as distributed generation capacity variations, load updates, and topology changes, they struggle to fully characterize the islanding state and are prone to feature invalidation and discrimination errors.

**Table 1:** Comparison of islanding detection methods based on intelligent algorithms.

References	Algorithm	Innovation	Advantages	Limitations
[15]	LSTM	Thirteen time-domain features are extracted from three-phase voltage at PCC, and six key features are selected for classification using LSTM algorithm.	High detection accuracy of 99.68%, short detection time of 72 ms, and strong noise immunity.	Relies on high-quality voltage signals and requires manual feature selection.
[16]	WT-CNN	Converts time-series signals into scalogram images via continuous wavelet transform, and uses CNN for image classification.	Image-based features with good intuitiveness.	Relatively low detection accuracy of 95.4%; relies on wavelet basis selection.
[17]	SDFT-EMD-LSTM	Combines sliding-window discrete Fourier transform, empirical mode decomposition, and attention-mechanism optimized LSTM.	Detection accuracy of 98.45%, detection time of 66.94 ms; strong time-series modeling, good anti-noise performance.	Complex feature engineering and model structure; feature extraction relies on manual design.
[18]	DWT-DNN	Extracts statistical features of voltage sequence components via wavelet transform, input to DNN for classification.	Detection accuracy of 99%, with comprehensive feature extraction covering multiple operating conditions.	Complex network structure, and high training cost.
[19]	FFT-KNN	Extracts positive, negative, zero sequence features of voltage, current and second harmonics, which input to KNN classifier.	High detection accuracy of 99%, short detection time of 5–15 ms, and no complex training required.	Features are manually designed; KNN performance degrades with largescale datasets; sensitive to second harmonics, which may lead to misclassification.
[20]	PMU-GNB	Extracts voltage angle, slip angle, acceleration angle via PMU; Gaussian Naive Bayes for islanding classification.	Simple feature extraction, no threshold calibration; short detection time of 0.179 ms.	Detection accuracy of 94.29%; GNB sensitive to noise.
[21]	μ-PMU-FT-RF	Uses micro-PMU to monitor zero or negative sequence voltage phasors, and calculates phase-angle sum as the detection index.	Low communication dependence with μ-PMU, short detection time of 20 ms.	Threshold determination requires extensive simulation; poor adaptability to weak features in NDZs.
[22]	PMU-ANN	PMU extracts voltage phasor, frequency, and ROCOF features, which are fed into ANN to classify islanding and non-islanding events.	Extremely high detection accuracy of 99.05%, average detection time of 185 ms, fast detection speed, and no NDZ.	Relies on PMU hardware support, and high cost.

Some studies [20–22] have attempted to introduce phasor measurement unit (PMU) to achieve time-synchronized phasor measurement, enabling high-precision and high-accuracy acquisition of electrical phasor information such as voltage magnitude, phase, frequency, and rate of change of frequency (ROCOF) at the point of common coupling. The dynamic differences of synchronized phasors are utilized to improve the response speed and discrimination accuracy of islanding detection. Simulations have been carried out on the IEEE standard node test system, verifying that the PMU-based islanding detection method features faster detection speed and shorter detection time. However, transient processes in the distribution network, such as voltage sags, three-phase unbalance, load switching, and harmonic interference, tend to cause phasor distortion, resulting in weak anti-disturbance capability and remaining detection blind spots under power balance conditions. Meanwhile, existing PMU-based detection methods still require manually designed discriminant features such as sequence components and phase angle differences, exhibiting insufficient generalization performance when system topology, power supply capacity, and load parameters change, and are difficult to adapt to complex scenarios with the grid integration of multiple types of power sources [23,24].

To address the above limitations, this paper proposes an islanding detection method based on STFT and CNN. STFT can effectively mine the local time-frequency features of time-series signals and generate time-frequency spectrograms, enhancing the identifiability of weak features within the non-detection zone. CNN extracts multi-dimensional features in both time and frequency domains from time-frequency spectrograms via convolution kernels. By integrating the correlations among multiple weak features, it automatically learns and constructs high dimensional decision boundaries, thereby achieving accurate classification between islanding and non-islanding states.

The main contributions of this paper are summarized as follows:

1. The fusion of STFT and CNN effectively solves the problems of insufficient robustness, feature invalidation, and classification deviation encountered by existing methods in complex multi-machine grid-connected distributed generation systems.
2. Simulation results verify that the proposed method can significantly improve detection accuracy under complex scenarios, providing a new technical approach to reduce the non-detection zone.

The remainder of the paper is organized as follows: Chapter 2 describes the proposed islanding detection method; Chapter 3 introduces the simulation setup and dataset generation process; Chapter 4 analyzes the experimental results and compares them with existing methods; Chapter 5 concludes the entire work.

## Islanding detection method

### Data collection phase

**Fig 1:**
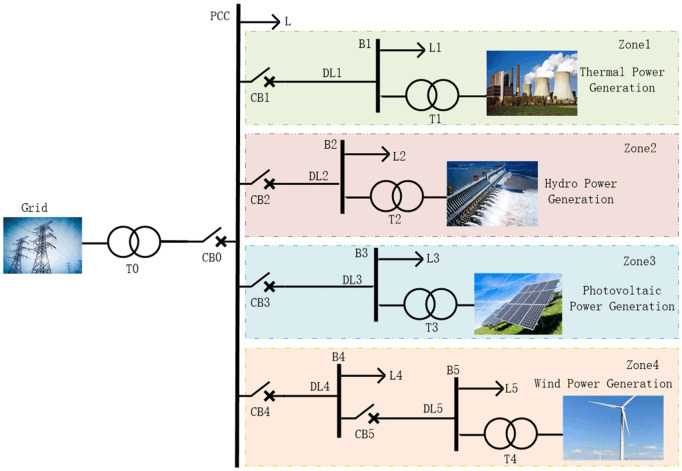
Schematic diagram of distributed generation grid-connected complex system.

As shown in Fig 1, distributed generation grid-connection complex system is built based on IEC 61850-7-420 standard [25,26]. This system consists of 4 distributed generations (DGs), including synchronous generation (region 1 is thermal power generation, region 2 is hydropower generation) and inverter-based generation (region 3 is photovoltaic power generation, region 4 is wind power generation). Table 2 fully lists the key parameters of DG1 to DG4, including rated capacity (MVA), rated voltage (kV), active/reactive load (MW/MVar), and transformer voltage ratio (kV). To comprehensively simulate the system operation, two major operating states of non-islanding and islanding are simulated. For non-islanding condition, the focus is on simulating situations where the system is prone to be in detection blind zones, including single-line-to-ground (SLG) faults, double-line-to-ground (DLG) faults, three-line-to-ground (TLG) faults, and load changes, etc. Islanding condition is realized by disconnecting different circuit breakers such as CB0 to CB5. The three-phase voltage and current waveforms at each node under both islanding and non-islanding conditions are acquired, and time series are generated and exported to MATLAB workspace to provide data support for subsequent analysis.

**Table 2:** Detailed parameters of distributed generation grid-connected system.

Region	Type	Power Rating	Voltage Rating	Load	Transformer	Instruction
**Grid**	Grid	20 MVA	120kV	1.5 MW /0.6 MVar	120/25 kV	Grid
**region 1**	thermal power generation	4 MVA	2.4kV	1.5 MW /0.3 MVar	2.4/25 kV	Synchronous DG
**region 2**	hydropower generation	4 MVA	2.4kV	1MW /0.2 MVar	2.4/25 kV	Synchronous DG
**region 3**	photovoltaic power generation	3 MVA	311 V	3MW /0.6 MVar	0.311/25 kV	Inverter DG
**region 4**	wind power generation	3 MVA	311 V	0.5MW /0.1 MVar 1MW /0.3MVar	0.311/25 kV	Inverter DG

### Data processing phase

**Fig 2:**
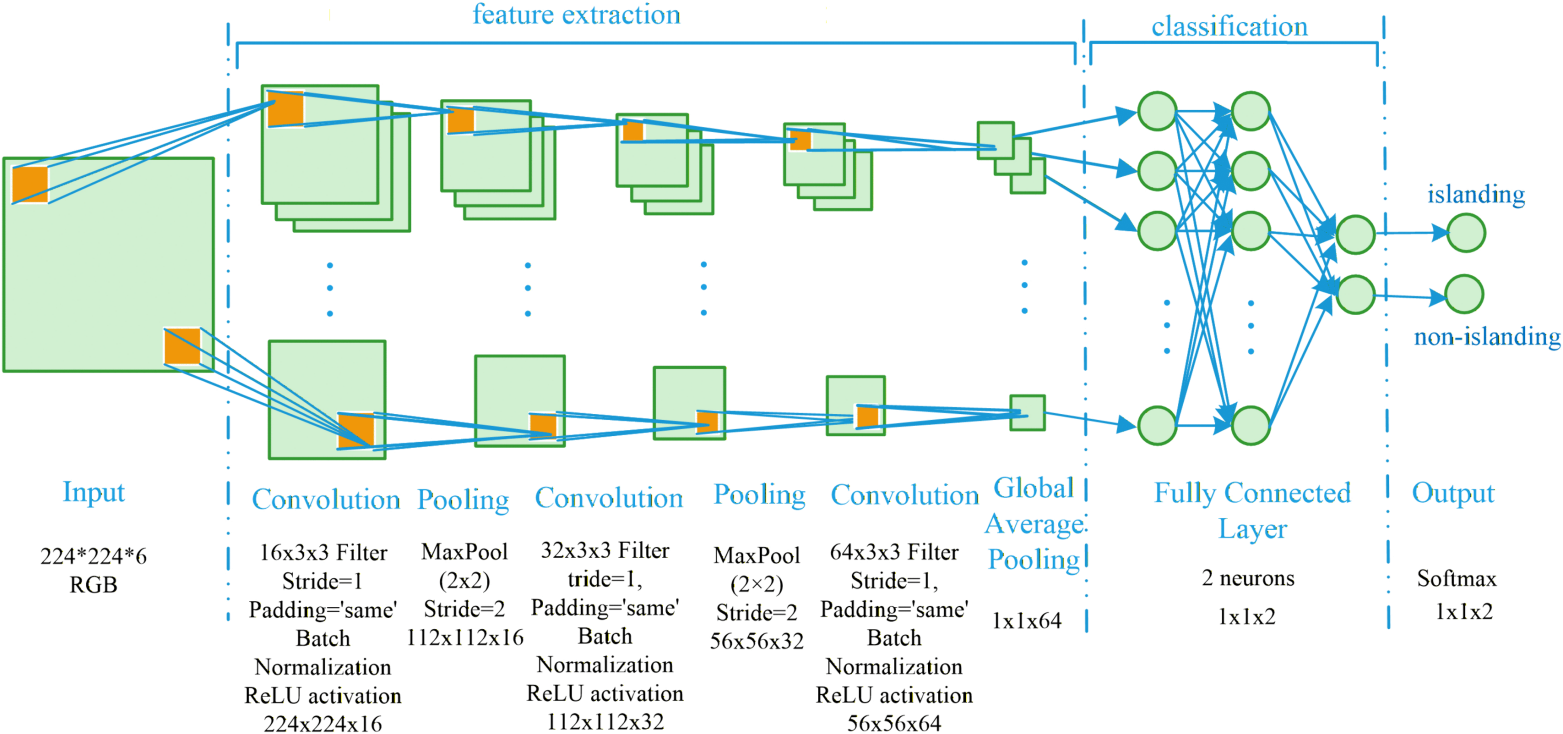
Network structure diagram of CNN.

As shown in Fig 2, perform preprocessing of framing and windowing on timeseries signals of voltages and currents at each node. The framing process cuts the long time-series signal into several overlapping or non-overlapping micro-frames by setting a time window with fixed duration. Windowing process applies a window function with smooth attenuation characteristics to both ends of the signal within each frame to suppress abrupt changes in the signal at frame boundaries, reduce the spectrum leakage caused by framing operation, and enhance the continuity of signal within frame to ensure the integrity of local signal features. After the preprocessing is completed, the built-in function of spectrogram () is called to execute STFT on each windowed frame signal. The specific parameters of STFT are shown in Table 3.The essence of STFT is to realize local spectrum analysis of non-stationary signals by introducing an analysis window sliding over time, obtain the spectral characteristics of each frame, and finally generate a time-frequency spectrogram reflecting the dynamic changes of frequency over time [27].

**Table 3:** STFT parameters.

Category	Parameter	Description
**Window Function**	Hamming window	*ω*_*(n)*_ = 0.54–0.46 * cos(2π*n*/(N-1)), where *n* = 0,1,...,N-1, N is the window length.
**Window Length**	256 points	Time length is 256/3840 = 0.06667 s
**Sampling Frequency**	3840 Hz	Satisfies Nyquist criterion
**FFT Points**	360 points	360 points can be factorized as 2^3^*3^2^*5
**Overlap Points**	192 points	75% overlap
**Time Resolution**	16.67 ms	64/3840 ≈ 0.01667 S
**Frequency Resolution**	10.67 Hz	*F*_*s*_/*n*_*FFT*_ = 3840/360 ≈ 10.67 Hz

The traditional Fourier transform can only obtain the overall frequency components of a signal within the entire time domain, and cannot characterize the instantaneous variation of frequency components over time, thus making it difficult to apply to non-stationary signal analysis. Through the localization processing of window function, STFT realizes the representation of signals on time-frequency two-dimensional plane [28]. STFT can effectively capture the time-varying characteristics of frequency components in non-stationary signals and has significant advantages in detecting transient characteristic signals such as frequency mutations and harmonic fluctuations during islanding detection [29].

By introducing time window, STFT captures the local frequency characteristics of the signal and obtains the dynamic characteristics of the signal changing over time. The generated time-frequency spectrograms is illustrated using a color heat map, where the horizontal axis corresponds to the time dimension, the vertical axis represents frequency information, and the depth of color reflects the signal energy intensity at the time-frequency point, facilitating the capture of characteristics such as frequency mutation points and harmonic injection when islanding occurs [30].

Extract the time-domain signals of three-phase voltages *V*_*a*_, *V*_*b*_, *V*_*c*_ and three-phase currents *I*_*a*_, *I*_*b*_, *I*_*c*_ from IDT simulation results. The signals are collected at a sampling rate of 3840 Hz, with a total duration of 0.75 s, resulting in 2880 points. STFT is used to convert the time-domain signals of the three-phase voltages and currents into time-frequency spectrograms. The STFT parameters are set as follows: 256-point Hamming window, 192-point overlap, and 360-point FFT. After generating the spectrogram for each phase signal, the maximum amplitude value among the three phases is taken as the representative value of that time-frequency point to obtain a time-frequency matrix, thereby converting the three-dimensional signal into a two-dimensional time-frequency image. This can highlight the maximum energy components among the three phases, which is helpful for detecting anomalies. The images are displayed on a dB scale, with the frequency range limited to 0–1000 Hz and the dynamic range set to −60 to 20dB. These images are saved as high-resolution PNG files with 300dpi, providing input data for the subsequent CNN islanding detection model.

To further highlight islanding features, it is necessary to perform denoising and filtering processing on time-frequency spectrograms to suppress background noise and interference signals, optimize image quality, and make islanding features easier to identify. For the time-series signals of voltage and current under different operating conditions of islanding and non-islanding, the above processing procedure is repeatedly executed to construct a standardized time-frequency spectrogram atlas, providing high-quality feature datasets for the subsequent training of islanding detection algorithms.

### Islanding detection phase

The spatial feature extraction capability of CNN model for time-frequency spectrograms is utilized to identify changes in harmonic distribution under islanding and non-islanding state. The network structure of CNN algorithm is shown in Fig 2, which is divided into input layer, convolution layer, pooling layer, fully connected layer, and output layer.

After input layer receives the time-frequency spectrograms, feature processing is completed through the alternating stacking of convolution layers and pooling layers. The specific process is as follows. First, in order to achieve feature alignment, all input images of voltage time-frequency spectrum and current time-frequency spectrum are resized to 224 × 224 × 3 and then concatenated into a single tensor of size 224 × 224 × 6 [30].

The convolution layers extract local features of the time-frequency spectrograms. Through multiple convolution kernels sliding from left to right and top to bottom on the time-frequency spectrograms, the inner product of local region and convolution kernel is calculated. Convolution kernel cover all channels of the input. Efforts are made to extract frequency bands strongly related to islanding state, such as frequency offsets in low-frequency bands and harmonic mutation variables in high-frequency bands, to reduce redundant information. A total of 3 convolutional layers are used, with 3 × 3 filters adopted for the convolutional layers, the padding method set Padding as ‘same,’ and the ReLU activation function applied. The number of filters is set to 16, 32 and 64 respectively to extract complex features.

Pooling layers reduce the parameter scale and computational load by shrinking the size of feature maps, enhance robustness to translation and scaling of time-frequency spectrograms, and achieve dimension reduction and anti-interference. Max pooling is applied to the first two convolutional layers with a pooling size of 2 × 2 for dimensionality reduction. Global pooling is adopted for the last convolutional layer with a pooling size of 1 × 1 × 64 to mitigate the risk of overfitting.

Then, the fully connected layers fuse the extracted local features into global features, flatten the multi-dimensional feature maps output by the pooling layers into one-dimensional vectors, and realize global feature fusion through full connection between weight matrices and neurons. Finally, the output layer generates the results and probabilities of islanding detection [31].

The time-frequency spectrum atlas is divided into a training-validation set and an independent test set at a ratio of 7:3. Due to the small sample size, it falls into the category of small-sample data. Therefore, the training-validation set is trained using five fold cross-validation to ensure that each sample participates in both training and validation. The specific operation is as follows: after randomly shuffling the time-frequency spectrogram atlas, it is evenly divided into five approximately equal-sized and non-overlapping fold. Then the model is train five times, each time using four folds for training and one fold for validation, with a different fold used for validation each time for cyclic validation. To avoid time waste caused by over-training, the maximum number of epochs in each fold training process is set to 10. After the training is completed, the average value of five fold results are finally taken as the final evaluation index of the model. The model with the best performance in five-fold training is selected, and its parameters are saved to the file of bestModel.mat. By inputting time-frequency spectrograms to be detected into this optimal model, the classification of islanding and non-islanding states can be completed [32]. The independent test set is input into the optimal model for islanding detection, and the results are compared with the true states to calculate the accuracy of the islanding prediction model, thereby measuring the generalization ability of the trained model.

**Fig 3:**
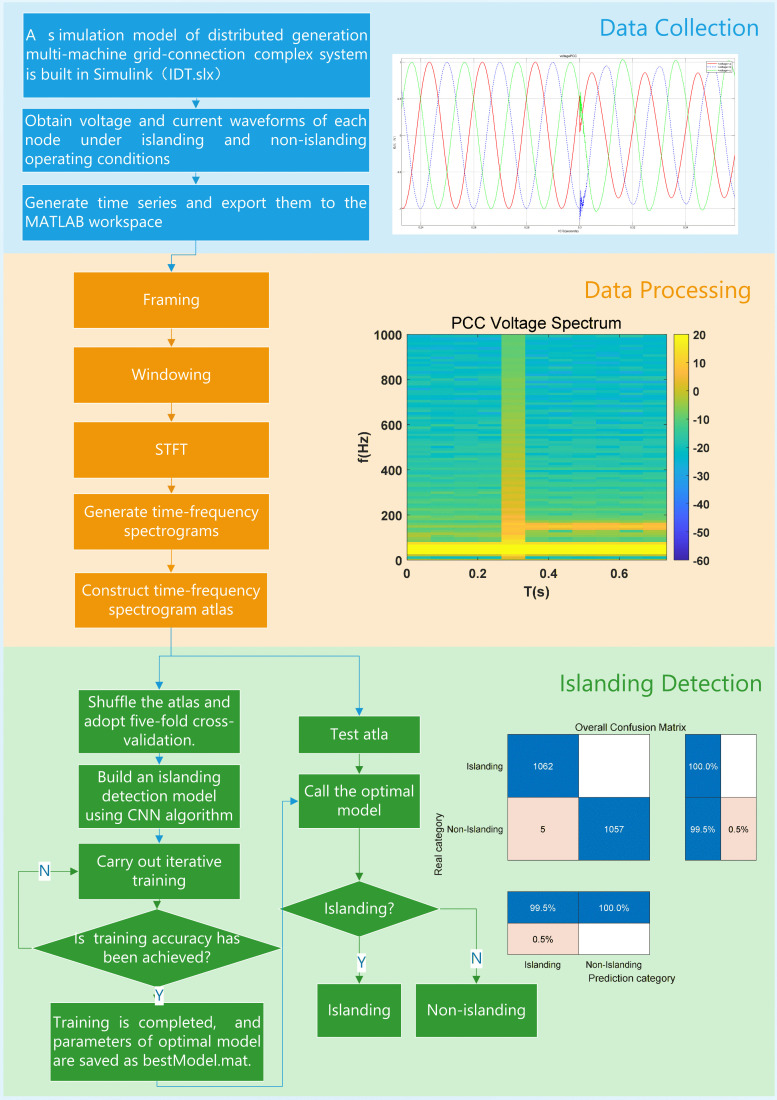
Flowchart of islanding detection.

The proposed islanding detection technology performs time-frequency spectrum analysis and calculation based on STFT to generate time-frequency spectrograms. After completing the extraction of feature data, CNN algorithm is used for training, and finally the optimal islanding detection model is obtained. Islanding detection is realized through the entire chain, and the specific flowchart is shown in Fig 3. Although the islanding detection method based on intelligent algorithms can significantly improve detection accuracy, it relatively increases determination time. Considering the handling of time urgency in relevant protection work [33–36], islanding detection can be set with a final time window guard of 1.8 s.

### Experimental verification

**Fig 4:**
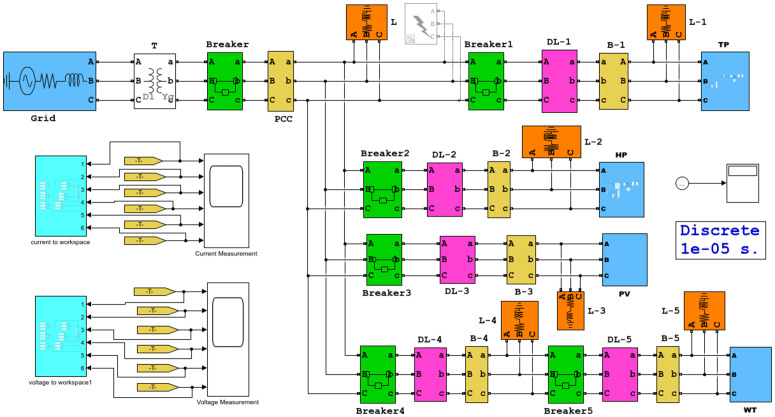
Simulation model of distributed generation grid-connected complex system.

As shown in Fig 4, a simulation model of distributed generation grid-connection complex system was built in MATLAB/Simulink. Non-islanding cases were simulated by single-line-to-ground (SLG) faults, double-line-to-ground (DLG) faults, three-line-to-ground (TLG) faults, and load changes. and islanding cases were simulated by disconnecting CB0-CB5. The voltage and current waveforms at each node of the system under both islanding and non-islanding conditions were simulated, and time series were generated and exported to MATLAB workspace. During simulation, the sampling frequency was set to 10 kHz, and a total of 2124 groups of sample datasets were finally collected, as shown in Table 4.

**Table 4:** Time-frequency spectrum detailed list.

Spectrum Atlas	System State	Quantity	Spectrum Atlas	System State	Quantity
Islanding by changing ∆P∆Q from-25% to 25%	Islanding	456	L load switching (3–12 MVA)	Non-islanding	60
Non-detection blind zone islanding	Islanding	366	L1 load switching（1–4MVA）	Non-islanding	60
Power balance	Islanding	120	L2 load switching（0.5–4MVA）	Non-islanding	60
Cross compensation	Islanding	120	L3 load switching（1–3MVA）	Non-islanding	60
Single-line-to-ground fault with short-circuit impedance 0.1–100 Ω	Non-islanding	240	L4 load switching（0.5–2MVA）	Non-islanding	42
Double-line-to-ground fault with short-circuit impedance 0.1–100 Ω	Non-islanding	240	L5 load switching（0.1–1MVA）	Non-islanding	60
Three-phase symmetrical short-circuit fault with short-circuit impedance 0.1–100 Ω	Non-islanding	240	Total	/	2124

### Simulation of islanding states

In the distribution lines, islanding conditions in regions 1–4 were simulated by disconnecting circuit breakers CB1–4, and 1062 sets of time-frequency spectrogram atlas under such conditions were obtained. Particular emphasis should be placed on simulating several common types of typical detection blind spots.

(a) Power balance between generation and load

A perfect islanding state with power balance is formed when the active power P_DG_ of the distributed generation is almost equal to the active power P_L_ of load, and the reactive power Q_DG_ is also approximately balanced with Q_L_. At this time, parameters such as voltage, frequency, current, and harmonics at the monitoring point remain almost stable without obvious fluctuations exceeding the threshold, leading to the failure of passive detection methods. For active detection, since the output power of DGs can smooth out active disturbances, the disturbances cannot accumulate to the detection threshold, which also causes the failure of active detection methods. This kind of islanding state where power generation is almost equal to load eliminates the power imbalance between DGs and load, making neither parameter mutation in passive detection nor disturbance accumulation in active detection occur. As a result, existing mainstream detection methods lose their judgment basis, becoming a typical detection blind zone and a key issue to be solved in the research on grid-connection safety of distributed generation [37].

(b) Cross compensation in multi-DGs coordinated operation

When grid is powered off, multiple DGs such as photovoltaic power generation, wind power generation, thermal power generation, and hydropower generation jointly match load through power complementarity, forming multi-source coordinated islanding state. At this time, power fluctuation of a single DG is compensated by other DGs, the overall voltage and frequency of system are approximately stable. Actively injected disturbances are jointly diluted by multi-source collaboration, making it difficult for existing detection methods to identify [38].

**Fig 5:**
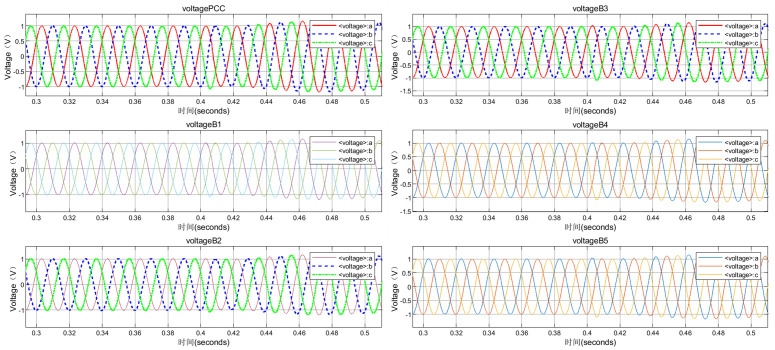
Voltage waveforms at each node under cross compensation.

**Fig 6:**
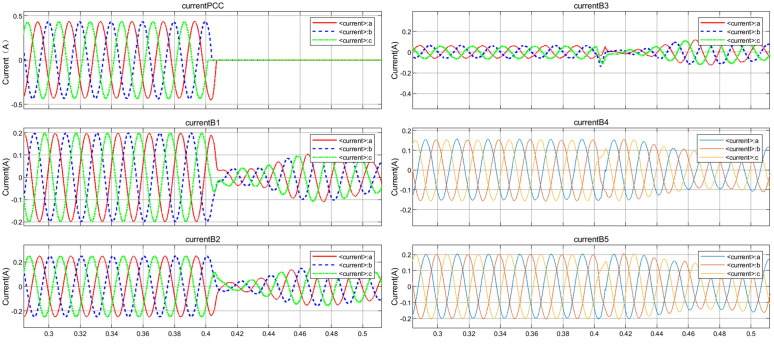
Current waveforms at each node under cross compensation.

**Fig 7:**
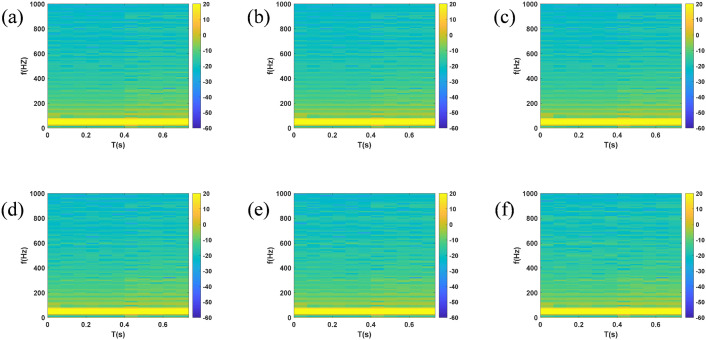
Spectrogram of voltage at each node under cross compensation (a) PCC (b) B1 (c) B2 (d) B3 (e) B4 (f) B5.

**Fig 8:**
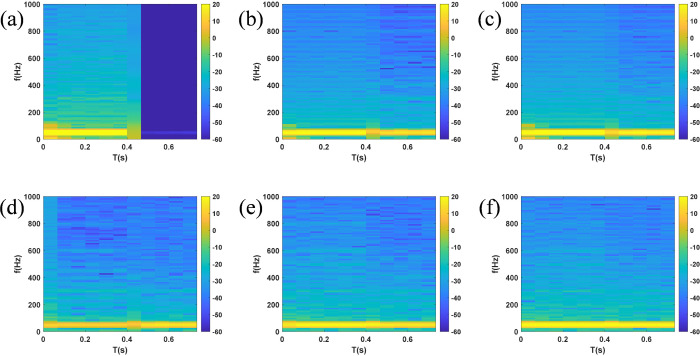
Spectrogram of current at each node under cross compensation (a) PCC (b) B1 (c) B2 (d) B3 (e) B4 (f) B5.

Taking the disconnection of circuit breaker CB0 at PCC at 0.4 s as an example to stop the power supply from the grid, so as to simulate the detection blind zone scenario of cross compensation in multi-DGs coordinated operation. The simulated voltage and current waveforms of each node are shown in Figs 5 and 6. After generating time series and exporting them to the MATLAB workspace, time-frequency spectrograms were generated by STFT, as shown in Figs 7 and 8. The time-frequency spectrogram of PCC voltage shows little change, which is the result of cross compensation caused by coordinated operation of multiple distributed generations. It can be seen from time-spectrogram of PCC current that both the amplitude and frequency of the spectrum change significantly after 0.4 s. The yellow spectrum components disappear, amplitude decreases, and dark blue spectrum appears at the same time, at which point PCC current reverses.

### Simulation of non-islanding states

**Fig 9:**
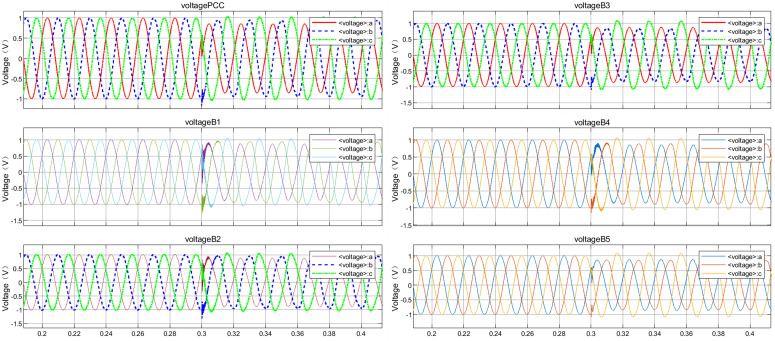
Voltage waveforms at each node under SLG fault.

**Fig 10:**
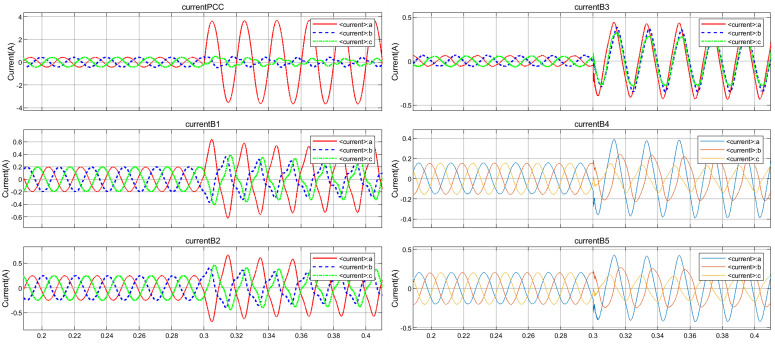
Current waveforms at each node under SLG fault.

Different fault conditions such as SLG, DLG, TLG at different lines and load changes of L1-L5 were simulated on distribution system, resulting in 1062 time-frequency spectrogram atlas under non-islanding condition. Taking the phase A single-line-to-ground fault occurring at 0.3 s on distribution line 1 in region 1 as an example to simulate non-islanding state, the voltage and current waveforms of each node obtained from the simulation are shown in Figs 9 and 10. It can be observed that the voltage and current waveforms both exhibit obvious distorted burrs at 0.3 s, the three-phase symmetry is destroyed, and the zero-crossing timing and peak interval all change. The time series generated from the voltage and current of each

**Fig 11:**
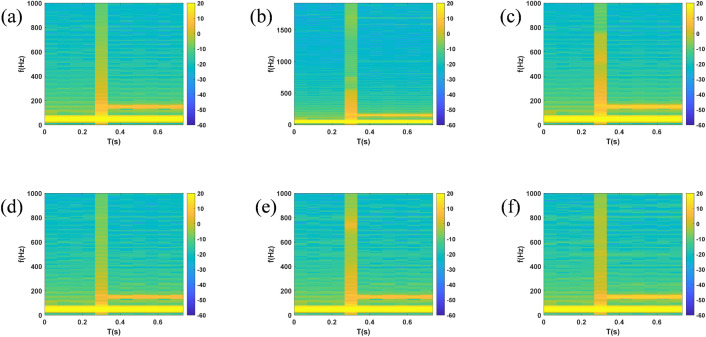
Spectrogram of voltage at each node under SLG fault (a) PCC (b) B1 (c) B2 (d) B3 (e) B4 (f) B5.

**Fig 12:**
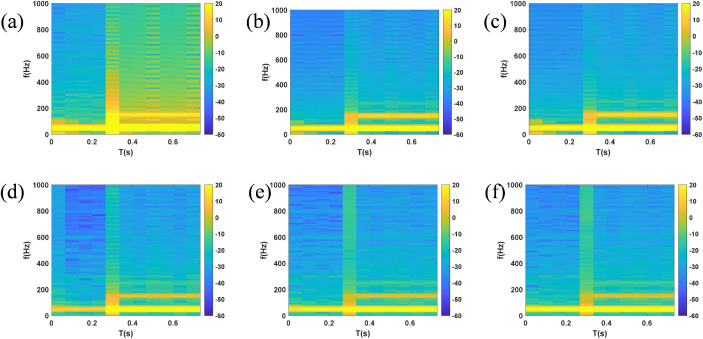
Spectrogram of current at each node under SLG fault (a) PCC (b) B1 (c) B2 (d) B3 (e) B4 (f) B5.

Node were exported to MATLAB workspace, and time-frequency spectrograms were generated via STFT, as shown in Figs 11 and 12**.** It can be seen from the bright green frequency bands in Fig 11 that the voltage spectrum of each node is dominated by 50 Hz power frequency. After single-line-to-ground fault occurs at 0.3 s, yellow-orange distributions appear on both sides of the power frequency in spectrogram, and the power frequency component exhibits frequency drift. In Fig 12, the bright green frequency band of PCC current increases sharply at 0.3 s, reflecting a several-fold increase in the amplitude of the power frequency current. Due to the asymmetry of single-line-to-ground fault, multi-frequency energy of fault phase current in current spectrogram at B1 node is significantly higher than that of non-fault phases.

## Results analysis

**Fig 13:**
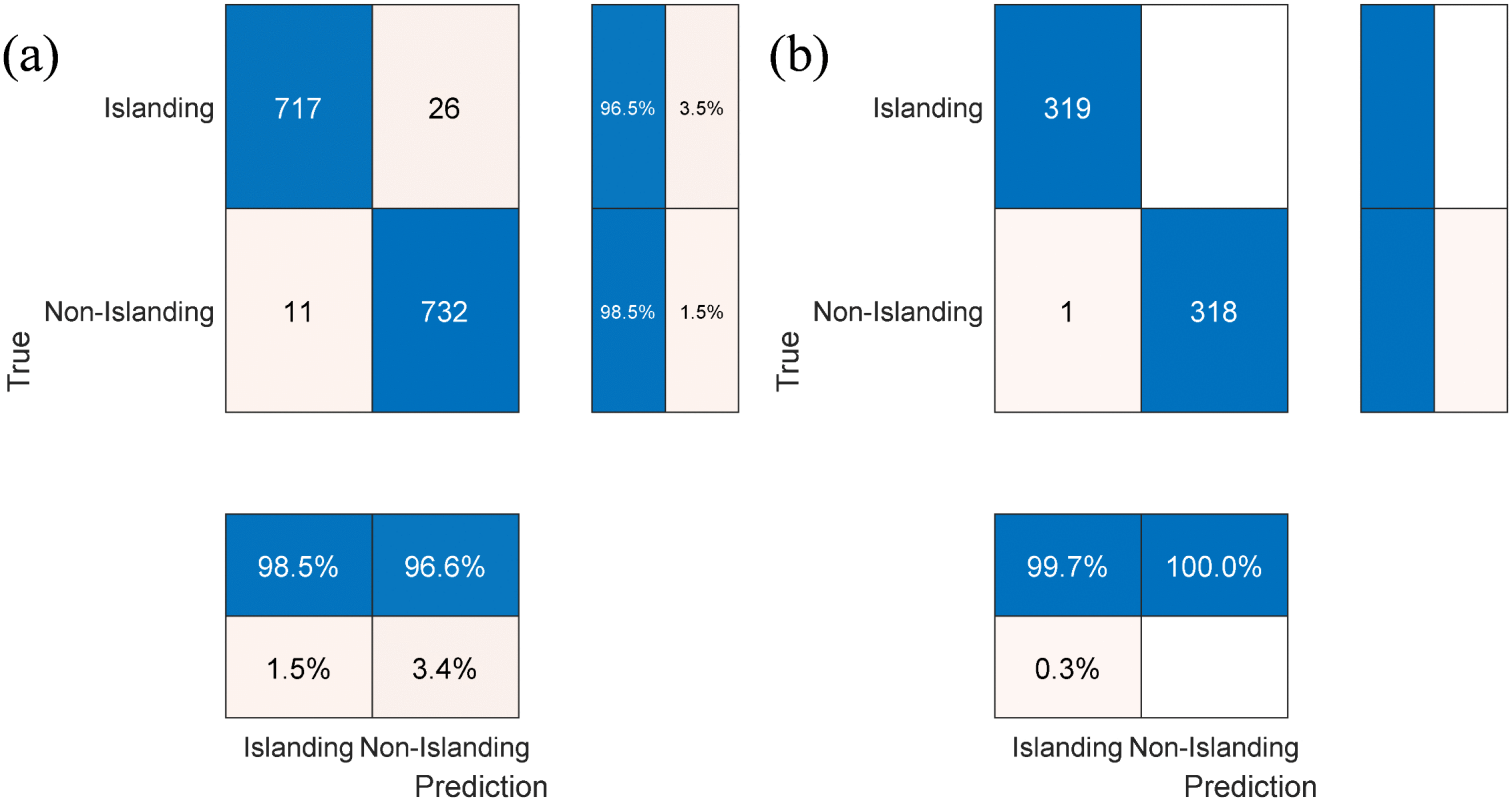
Confusion matrix (a) five-fold cross validation (b) test set.

After the training of the proposed islanding detection model, the confusion matrix of the islanding detection model was obtained, as shown in Fig 13.

The five-fold cross-validation was implemented on a dataset with a total of 1486 samples, where the actual islanding and non-islanding cases were equally divided into 743 samples each. For the actual islanding samples, 26 instances were misclassified as non-islanding states, resulting in TPA of 96.5%. For the actual non-islanding samples, only 11 instances were misclassified as islanding states, corresponding to TNA of 98.5%. In terms of the overall cross-validation performance, the model achieved an overall prediction accuracy of 97.5%, with FPR of 1.5% and FNR of 3.4%. These metrics collectively indicate that the proposed model possesses robust islanding identification capability and a low misclassification risk in the validation phase.

Subsequently, the model was evaluated on an independent test set containing 638 samples that were completely separated from the training and validation datasets and not involved in any model training process. All actual islanding samples in the independent test set were correctly identified, and only one actual non-islanding sample was misclassified as an islanding state. This led to an overall prediction accuracy of 99.8% and an ultra-low FPR of 0.3% for the model on the unseen data, which verifies that the model can maintain high classification accuracy on data outside the training set.

**Fig 14:**
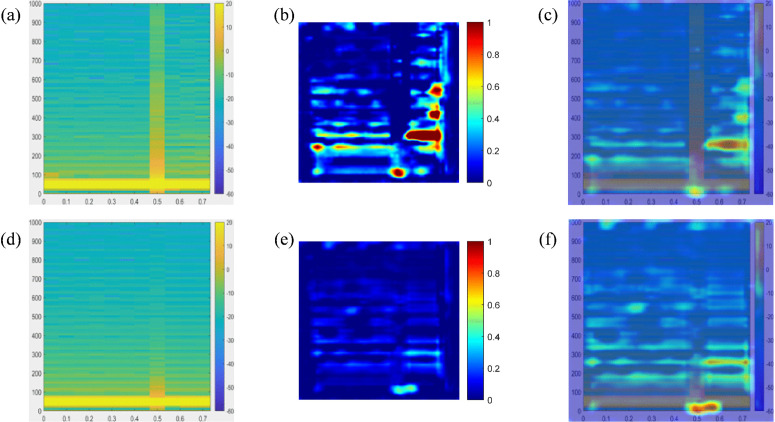
Triple-view Grad-CAM heatmap (a) Voltage spectrogram (b) Grad-CAM heatmap of voltage (c) Voltage overlay (d) Current spectrogram (e) Grad-CAM heatmap of current (f) Current overlay.

For false positive islanding misclassification cases in the test set, triplet heatmaps of voltage and current signals were generated, as shown in Fig 14. The original voltage time-frequency spectrum exhibits no typical persistent medium-to-high frequency harmonic enhancement bands characteristic of islanding events, with only local low-frequency energy fluctuations. The red-yellow highlighted regions in the Grad-CAM voltage heatmap are mainly in the low-frequency band of 200–300 Hz, accompanied by a small number of scattered discrete activation points in the medium-to-high frequency range, without continuous large area activation bands. The overlaid plot verifies the correspondence between the activation regions and the low-frequency fluctuations in the time-frequency spectrum. When a distributed generation unit loses support from the main grid, voltage and current signals exhibit frequency drift, low-frequency harmonic distortion, and slow amplitude fluctuations, but no obvious high-frequency transient disturbances. However, load switching and grid disturbances in non-islanding scenarios can also cause low-frequency voltage fluctuations, which overlap with the low-frequency drift characteristics of islanding, leading the model to mistakenly identify noise as islanding features. The Grad-CAM heatmap of the current signal also shows a scattered weak activation pattern, failing to provide effective strong discriminative information. Grid noise interference introduces confounding features, which become a source of misclassification. The model overfocuses on low-frequency voltage fluctuation features in non-islanding scenarios, causing it to incorrectly identify disturbance noise as an islanding condition.

In summary, the proposed model achieves an overall accuracy of over 97% in both five-fold cross-validation and independent testing, with excellent identification performance for both islanding and non-islanding states, and a further improved accuracy on the independent test set. These results fully confirm that the model is free from overfitting and exhibits superior generalization ability.

**Fig 15:**
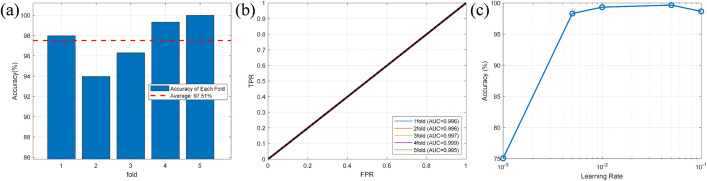
Parameters of each fold (a) Accuracy of each fold (b) ROC curve (c) Sensitivity analysis.

A 5-fold cross-validation strategy was adopted for the CNN training process, with a maximum of 10 training epochs set for each fold and 530 iterations per epoch. As shown in Fig 15a, the accuracy of the 5-fold cross-validation is approximately 97.5%, 96.0%, 96.5%, 98.0%, and 99.5%, respectively. The average accuracy across all 5 folds is 97.5%. The accuracy of each fold exceeds 96%, which indicates that the model has a strong recognition capability during the cross-validation stage. The accuracy of the 2nd fold is the lowest, at about 96.0%, while the accuracy of the 5th fold is the highest, at approximately 99.5%. The detailed performance metrics of the 5-fold cross-validation are presented in Table 5. To understand the source of the fluctuation, we analyzed the data distribution in each fold. The sensitivity of the model is not random but related to specific operating conditions. The fold with the lowest accuracy of 96.0% contained an excessively high proportion of challenging conditions, such as light load, high harmonic background, and high load quality factor. These conditions are particularly difficult for islanding detection. In contrast, the fold with the highest accuracy of 99.5% mainly consisted of simple operating conditions, where islanding features are easier to identify. This indicates that the model’s accuracy decreases slightly under extreme conditions but remains above 96% overall. Therefore, the lower bound of performance should be prioritized in actual deployment.

**Table 5:** Detailed indicators for five-fold cross-validation.

Fold	Accuracy	AUC	Precision	Recall	F1-Score
**1**	97.50%	0.996	98.88%	95.95%	0.9740
**2**	96.00%	0.996	96.45%	95.28%	0.9587
**3**	96.50%	0.997	96.62%	95.97%	0.9629
**4**	98.00%	0.999	98.33%	98.33%	0.9833
**5**	99.50%	0.995	98.95%	98.80%	0.9887

**Fig 16:**
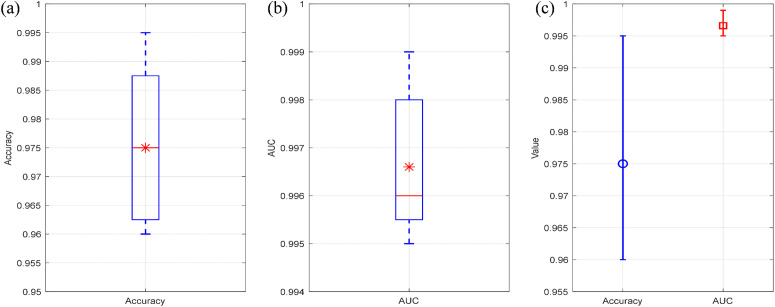
Performance metrics plot of five-fold cross-validation (a) Distribution of cross-validation accuracy (b) Distribution of cross-validation AUC (c) Error bar chart of performance metrics.

The accuracy of 99.84% on the independent test set is slightly higher than the average cross-validation accuracy of 97.5%, which further verifies the generalization ability of the model and effectively rules out spurious high performance caused by overfitting. Statistical analysis of the cross-validation results shows that the 95% confidence interval for the model’s accuracy is [0.9580, 0.9920], with minimal inter-fold variance, demonstrating that all performance metrics have high statistical reliability. The box plot of accuracy reveals that the accuracy of each fold ranges from 0.96 to 0.995 with a narrow box, which intuitively reflects the good stability of the model’s performance under different data partitions. The AUC box plot in Fig 16 shows that the average AUC of the model is approximately 0.9966 with an extremely narrow box, indicating a high level of classification consistency of the model across different data subsets. the analysis results of the error bar plot for performance metrics further confirm that the fluctuation ranges of both the model’s accuracy and AUC are at an extremely low level, which reaffirms the high statistical reliability of the experimental results and the absence of significant fold variance. As shown in Fig 15b, the ROC curves under five-fold cross-validation scenario exhibit an ideal upward trend with AUC values close to 1, indicating that the model can efficiently perform islanding detection with almost no classification errors. In addition, Fig 15c shows that the model has a reasonable sensitivity to hyperparameters, with the range of 10–2.5 to 10–1.5 identified as the optimal learning rate interval for the model. Within this interval, the model achieves the highest recognition accuracy with stable performance. This result further eliminates the possibility of spurious high experimental performance caused by trivial separability or overfitting, and fully verifies the practical feasibility of the proposed CNN model for islanding detection.

The performance of the proposed islanding detection method has been compared with other islanding detection methods, as shown in Table 6. The method achieves an accuracy of 99.84%, which is higher than that of other algorithms. The detection time of the proposed scheme is 0.121 seconds, which is longer compared to other algorithms but meets the requirement of 0.2 s specified in the IEEE 1547–2018 standard. In summary, compared with existing intelligent algorithms such as RNN, ANN, and LSTM, the proposed scheme exhibits the optimal performance in terms of accuracy and is more suitable for islanding detection scenarios requiring high detection precision.

**Table 6:** Comparison with other islanding detection methodology.

Methodology	Measurement Point	Accuracy	FNR	Detection Time	Reference
**RNN**	PCC	99.68%	0.32%	0.072 s	[10]
**ANN**	PCC	99.05%	0.95%	0.1 s	[22]
**LSTM**	PCC	98.4%	1.6%	0.067 s	[17]
**Proposed Scheme**	PCC	99.84%	0.16%	0.121 s	/

## Conclusions

To Solve the problem of islanding detection blind zones in complex multi-machine systems for distributed generation, this paper proposes an islanding detection model based on STFT and CNN algorithm. Voltage and current waveforms at each node under non-islanding and islanding operating conditions are obtained through model which is built in MATLAB/Simulink. After generating time series from these waveforms, time-frequency spectrums analysis is generated by STFT. Upon completion of feature data extraction, CNN algorithm is used for training and iteration to generate the optimal islanding detection model. Simulation results show that the proposed islanding detection method meets the time requirement of IEEE standards, achieves the detection accuracy of 99.84% on the independent test set, and obtains the missed detection rate of 3.4% under five-fold cross-validation, which significantly reduces the non-detection zone. This study provides theoretical support and engineering references for islanding detection in multi-machine grid-connected systems with high renewable energy penetration and complex load characteristics. In the future, we will explore transfer learning techniques to improve the robustness of the trained model against unknown topologies.
